# Transcriptomic differences noted in *Glaesserella parasuis* between growth in broth and on agar

**DOI:** 10.1371/journal.pone.0220365

**Published:** 2019-08-06

**Authors:** Samantha J. Hau, Kathy T. Mou, Darrell O. Bayles, Susan L. Brockmeier

**Affiliations:** 1 Virus and Prion Research Unit, National Animal Disease Center, ARS, USDA, Ames, Iowa, United States of America; 2 Oak Ridge Institute for Science and Education, Oak Ridge, Tennessee, United States of America; 3 Infectious Bacterial Diseases Research Unit, National Animal Disease Center, ARS, USDA, Ames, Iowa, United States of America; Università degli Studi di Pavia, ITALY

## Abstract

*Glaesserella parasuis* is the cause of Glӓsser’s disease in pigs and is a significant contributor to post-weaning mortality in the swine industry. Prevention of *G*. *parasuis* disease relies primarily on bacterin vaccines, which have shown good homologous protection and variable heterologous protection. Bacterin production involves large scale growth of the bacteria and proteins produced during the proliferation phase of production become important antigens that stimulate the immune response. In order to evaluate genes activated during *G*. *parasuis* growth on different media substrates, the transcriptome of broth and agar grown *G*. *parasuis* strain 29755 were sequenced and compared. The transcription of most purported virulence genes were comparable between broth and agar grown *G*. *parasuis*; however, four virulence-associated genes, including *ompA* and *vapD*, had elevated expression under agar growth, while six virulence-associate genes had elevated expression during broth growth, including several protease genes. Additionally, there were metabolic shifts toward increased protein and lipid production and increased cellular division in broth grown *G*. *parasuis*. The results contribute to the understanding of how growth substrate alters gene transcription and protein expression, which may impact vaccine efficacy if immunogens important to the protective immune response are not produced under specific *in vitro* conditions. While the results of this work are unable to fully elucidate which growth medium presents a transcriptome more representative of *in vivo* samples or best suited for bacterin production, it forms a foundation that can be used for future comparisons and provides a better understanding of the metabolic differences in broth and agar grown bacteria.

## Introduction

*Glaesserella parasuis* (formerly *Haemophilus parasuis*) is a commensal of the swine nasal cavity and the causative agent of Glӓsser’s disease, a significant cause of post-weaning mortality in pigs [1]. To provide protection against systemic disease with *G*. *parasuis*, pigs are most often vaccinated with bacterins. Bacterins have shown good efficacy in homologous protection and variable results under heterologous challenge [2–4]. This is thought to be associated with serotype-specific immunity, but may also relate in part to gene expression and the specific immunogenic proteins produced by the bacterium during proliferation for bacterin production.

Growth substrate is known to affect gene expression [5], which may alter the presence of important immunogens and virulence factors on the bacterial cell surface and result in a less effective vaccine. To date, there are few reports investigating gene transcription in *G*. *parasuis* and these focus on mimicking *in vivo* conditions to detect genes of interest during infections [6, 7]. The effect that growth substrate has on gene expression in *G*. *parasuis* has not been investigated.

This study aimed to define the transcriptome of *G*. *parasuis* strain 29755 when grown in broth and on agar. The results were utilized to better understand the impact of growth substrate on transcription, the production of immunogenic proteins, and the potential impacts this may have on vaccine development. This study will also form the foundation for comparison with future work utilizing *in vivo G*. *parasuis* samples.

## Materials and methods

### Bacterial strains and culture conditions

Experiments were performed on *G*. *parasuis* 29755, a serotype 5 isolate cultured from the lung of a pig with Glässer’s disease and shown to be virulent in colostrum-deprived pigs [8, 9]. *G*. *parasuis* 29755 was grown in brain heart infusion (BHI) broth or agar plates supplemented with 0.01% nicotinamide adenine dinucleotide (NAD) and 5% heat-inactivated horse serum (BHI–NAD–HS) in the presence of 5% CO_2_ at 37°C.

### RNA extraction of *in vitro* grown *G*. *parasuis*

*G*. *parasuis* was grown for RNA extraction in BHI-NAD-HS broth and on BHI-NAD-HS agar with five replicates per growth substrate. Agar grown bacteria were prepared from an overnight lawn harvested in 1 mL of BHI-NAD-HS broth. Broth grown bacteria were prepared by inoculating BHI-NAD-HS broth with *G*. *parasuis* grown overnight on BHI-NAD-HS agar. The cultures were incubated for 6 hours to an adjusted OD_600_ between 0.42–0.44 (colony forming unit (CFU)/mL of 10^9^). To prepare RNA for extraction, cultures were pelleted at 3,220xg, supernatant was decanted, and TRI reagent (ThermoFisher Scientific, Waltham, MA) was added. Cultures were vortexed for 1 minute, incubated at room temperature for 5 minutes, then stored at -80°C until RNA isolation.

To extract total RNA from samples, chloroform was added in a 1:5 ratio to TRI Reagent-cell mixture. The mixture was centrifuged at 4°C for 15min at 13,000rpm and the aqueous phase was transferred. Ethanol was added in a 1:1 ratio to the aqueous phase and the RNA was processed using the miRNeasy Mini Kit (Qiagen, Germantown, MD) following manufacturer’s instructions. On-column DNase treatment was performed using the RNase-free DNase set (Qiagen). Following RNA extraction, 10μg of extracted RNA was further treated with the TURBO DNA-free kit (ThermoFisher Scientific) and RNA was quantified using the NanoDrop ND-2000 spectrophotometer (ThermoFisher Scientific). RNA quality was assessed using the Agilent 2100 Bioanalyzer RNA 6000 Nano kit (Agilent Technologies, Santa Clara, CA).

### RNAseq library preparation and sequencing

To remove residual prokaryotic rRNA, the DNA-free total RNA was treated with the Ribo-Zero Gold Epidemiology Kit (Illumina, San Diego, CA) according to manufacturer’s instructions. Removal of rRNA was assessed using the Agilent 2100 Bioanalyzer RNA 6000 Pico kit (Agilent Technologies). Samples were submitted to the Iowa State University DNA Facility in Ames, IA for library preparation using the Stranded Total RNASeq library preparation kit (Illumina). Libraries were sequenced on one flow cell using the Illumina HiSeq 3000 platform to generate 100-nucleotide single-end reads on high output mode.

### Transcriptomic analysis

The initial quality of the sequencing reads was assessed using FastQC [10]. Reads were adapter and quality trimmed using Skewer [11], and the post-trimming read quality was again determined with FastQC. The Bowtie aligner was used to map trimmed reads to the *G*. *parasuis* strain 29755 genome (NCBI accession CP021644.1) [12]. Samtools was used to convert the mapped reads to the format needed for counting [13]. The read counts per gene were calculated by using HTSeq-count to process the mapped read alignment files [14]. DESeq2 was used to perform the differential expression analysis [15]. The count file data for all the samples were transformed using a regularized log transformation and then analyzed by clustering and visualization of the clustering via principle component analysis (PCA) and multi-dimensional scaling (MDS) to determine whether any of the samples were outliers due to uncontrolled experimental errors. No outlier samples were indicated and all samples were subsequently used. The count data for the samples were loaded into DESeq2 for the full analysis, the contrasts of interest were specified, and the differential expression (fold log_2_ difference) was calculated. The difference testing was done to identify genes differentially expressed (P-value < 0.001, false discovery rate (FDR) 0.1%) more than 1.6 log_2_ fold change (i.e. approximately 3x fold arithmetic change).

The genes identified as differentially expressed were assigned, to the largest extent possible, to their respective clusters of orthologous genes (COGs) to determine which COGs were most affected by growth condition [16].

## Results

### Clustering of growth conditions

**Fig 1:**
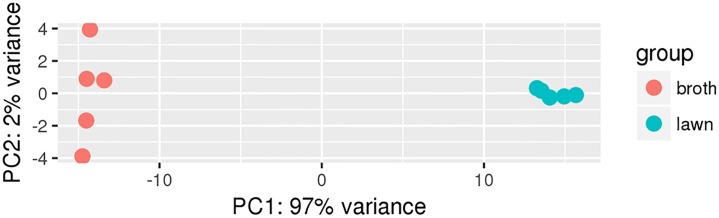
Principal component analysis (PCA) plot evaluating the variance between samples. The transcriptome of broth grown (orange) and agar grown (blue) *G*. *parasuis* replicates clustered within a treatment group (growth medium). The majority of variance detected was associated with differences between growth medium utilized.

A principal component analysis (PCA) plot of the transcriptional profile revealed highly similar profiles within a treatment group and clear separation between the transcriptome of *G*. *parasuis* under different growth conditions (Fig 1). The PC1-axis represented the majority of variance (97%), while small portion (2%) of the variance was represented by the PC2-axis. Replicates from the same treatment group clustered together, indicating most of the variance within the study was generated by differences associated with the growth substrate.

### Differentially expressed genes between plate and broth growth

Mapped reads totaled 25–50 million reads per sample. The results of differential expression analysis are presented in S1 Table. Genes found to have a statistically significant (adjusted P-value < 0.001) and greater than 1.6 log_2_ fold change in expression between *G*. *parasuis* grown in broth culture or grown on agar are listed in S2 Table. There were 231 genes found to be differentially expressed depending on the growth medium utilized, with 124 genes showing elevated expression in broth growth *G*. *parasuis* and 107 genes showing elevated expression when *G*. *parasuis* was grown on BHI-NAD-HS agar.

Previously identified virulence factors were evaluated for differential expression between growth conditions [9, 17–19] (S3 Table). Ten genes with a suspected role in virulence met the threshold of differential expression between broth and agar grown *G*. *parasuis*. Six genes had elevated expression under broth growth: phosphopantetheine adenylyltransferase, 3-deoxy-D-manno-octulosonic acid transferase, UDP-N-acetyl-D-mannosamine dehydrogenase, autotransporter outer membrane beta-barrel domain-containing protein, RIP metalloprotease *rseP*, and pitrilysin. Four genes showed elevated expression under agar growth: ADP-heptose-LPS heptosyltransferase, porin *ompA*, type II secreptory pathway pseudopilin (*pulG*) and virulence associated protein D (*vapD*). Other virulence genes, including the capsule locus genes, did not meet the threshold for differential expression when the growth conditions were compared (S3 Table).

### Cluster of orthologous group analysis

Cluster of orthologous group (COG) analysis identified the functional categories of differentially expressed genes between the two growth conditions. The results of COG analysis are in Table 1. In broth grown *G*. *parasuis*, there was greater representation of genes in the following COG classifications: amino acid transport/metabolism (E), nucleotide transport/metabolism (F), translation/ribosomal structure and biogenesis (J), replication/recombination/repair (L), cell wall/membrane/envelope biogenesis (M), and inorganic ion transport/metabolism (P). Agar grown *G*. *parasuis* had greater representation of genes in the COG classifications of carbohydrate transport/metabolism (G) and lipid transport/metabolism (I).

**Table 1:** COG analysis results.

OG_class	Number of Genes with Increased Expression during Broth Growth	Number of Genes with Increased Expression during Agar Growth	Total Number of Differentially Expressed Genes	Function Description
C	8	7	15	Energy production and conversion
D	1	0	1	Cell cycle control, cell division, chromosome partitioning
E	19	8	27	Amino acid transport and metabolism
F	10	3	13	Nucleotide transport and metabolism
G	2	9	11	Carbohydrate transport and metabolism
H	6	6	12	Coenzyme transport and metabolism
I	1	5	6	Lipid transport and metabolism
J	6	3	9	Translation, ribosomal structure and biogenesis
K	5	5	10	Transcription
L	10	3	13	Replication, recombination and repair
M	10	5	15	Cell wall/membrane/envelope biogenesis
O	9	8	17	Posttranslational modification, protein turnover, chaperones
P	10	4	14	Inorganic ion transport and metabolism
Q	0	1	1	Secondary metabolites biosynthesis, transport and catabolism
R	17	6	23	General function prediction only
S	9	9	18	Function unknown
T	4	5	9	Signal transduction mechanisms
U	2	1	3	Intracellular trafficking, secretion, and vesicular transport
-	10	29	39	Not in a COG

Further investigation into gene function indicated increased expression of genes in different metabolic pathways between broth and agar grown *G*. *parasuis*. Broth grown *G*. *parasuis* showed increased expression of genes involved in the utilization of nitrogen as a terminal electron acceptor, as opposed to agar grown *G*. *parasuis* which had increased expression of genes involved in the citric acid cycle and conversion of fermentation byproducts (COG C). Additionally, there was elevated expression of genes involved in protein and nucleic acid turnover in broth grown *G*. *parasuis* (COG O, L, and J).

## Discussion

Growth substrate has been shown to alter gene transcription [5]; however, this has been associated with changes in the media base from an enriched to a minimal medium. Differences in transcription may play a role in vaccine efficacy if one condition promotes greater production of immunoprotective antigens. To investigate gene transcription under different growth conditions and identify the growth substrate that provides a transcription profile more able to provide a protective immune response, we sequenced the transcriptome of *G*. *parasuis* 29755 when grown on BHI-NAD-HS agar and in BHI-NAD-HS broth.

Transcriptional differences were noted between broth and plate grown *G*. *parasuis*. Broth grown *G*. *parasuis* had greater expression of genes involved in DNA replication, cell wall synthesis, and cell division, indicating more rapid cellular proliferation. Additionally, broth grown *G*. *parasuis* had greater expression of genes involved in proteolysis, amino acid uptake, and amino acid synthesis, which are necessary to provide the amino acids utilized in cellular proteins. Expression of genes involved in protein and lipid synthesis were also elevated, which is likely associated with proliferation and production of cellular proteins and cellular membranes. It also appeared that broth grown *G*. *parasuis* may shift its metabolism to utilize nitrogen as an electron acceptor, which may indicate portions of the liquid culture become oxygen depleted during growth. Agar grown *G*. *parasuis* showed elevated expression of carbohydrate uptake and breakdown proteins, which may indicate agar as a substrate has less available carbohydrates and may necessitate greater resources being spent in energy acquisition.

It is difficult to speculate on which method of growth will generate a bacterin better capable of providing protective immunity. The transcription levels of purported *G*. *parasuis* virulence factors were comparable between both growth methods for the majority of virulence associated genes. Differentially expressed virulence genes were found in both growth conditions. Agar grown *G*. *parasuis* showed elevated expression of *ompA* and *vapD*. In other Gram negative bacteria, OmpA has been associated with adhesion, cellular infectivity and survival, and immune evasion [20]. Additionally, vaccination with OmpA has been associated with partial protection in some animal models of *G*. *parasuis* infection [21]. The *vapD* gene is thought to play a role in cellular infectivity based on the function of homologous genes in *Haemophilus influenzae* and *Rhodococcus equi* [22, 23]; however, its role in *G*. *parasuis* infection has not been confirmed. Broth grown *G*. *parasuis* had elevated expression several proteases, including a beta-barrel domain containing autotransporter with a putative serine protease function, *rseP*, and pitrilysin. Additionally, agar grown *G*. *parasuis* appear to be utilizing different metabolic pathways and may be replicating at a slower rate. Previous *in vivo* work identified reduced replication rates of *G*. *parasuis* during lung infection [6]. The bacteria had reduced expression of genes involved in translation and nucleotide metabolic processes [6], which is more consistent with *G*. *parasuis* grown on agar media than in broth. However, *G*. *parasuis* from the lung infection model also had reduced expression of genes involved in cellular carbohydrate metabolic processes, the citric acid cycle, and the electron transport chain [6]. These same COGs showed increased expression in agar grown *G*. *parasuis* in our study.

The data presented here indicate differences in transcription between *G*. *parasuis* grown on agar and in broth culture. Differences in gene transcription and protein production have the potential to alter the prevalence of important immunogens that may contribute to the protective immune response. At this time, we are unable to say which growth substrate generates a transcriptional profile most similar to that of *G*. *parasuis* during systemic infection, as we do not have *in vivo* data for analysis; however, this data can be utilized in future studies to help assess whether broth or agar growth most closely replicates *in vivo* conditions. The data generated in this study ultimately forms the foundation of future RNA sequencing work and will enable comparisons that may contribute to the generation of novel, more effective vaccines or the capacity to mimic *in vivo* conditions in the laboratory.

## Supporting information

S1 Table Differential expression results. The results output from the differential expression analysis comparing *G*. *parasuis* 29755 grown in broth and on agar.
(XLSX)

S2 Table Genes with significant differential expression. Genes from S1 Table with a statistically significant change of 1.6 fold or greater differential expression between *G*. *parasuis* 29755 grown in broth and on agar. Broth grown *G*. *parasuis* was utilized as the reference, with positive fold change indicating an increase in agar grown *G*. *parasuis* as compared to broth.
(XLSX)

S3 Table Differential expression results for virulence associated genes. Genes that have been previously associated with virulence were selected from the whole differential expression data (S1 Table) for comparison between the two treatment groups.
(XLSX)
